# Description of *Triatomahuehuetenanguensis* sp. n., a potential Chagas disease vector (Hemiptera, Reduviidae, Triatominae)

**DOI:** 10.3897/zookeys.820.27258

**Published:** 2019-01-28

**Authors:** Raquel Asunción Lima-Cordón, María Carlota Monroy, Lori Stevens, Antonieta Rodas, Gabriela Anaité Rodas, Patricia L. Dorn, Silvia A. Justi

**Affiliations:** 1 Department of Biology, University of Vermont, Burlington, Vermont, United States; 2 The A; 3 pplied Entomology and Parasitology Laboratory at Biology School, Pharmacy Faculty, San Carlos University of Guatemala, Guatemala; 4 Department of Biological Sciences, Loyola University New Orleans, New Orleans, Louisiana, USA; 5 Walter Reed Biosystematics Unit, Smithsonian Institution Museum Support Center, Maryland, USA; 6 Walter Reed Army Institute of Research, Silver Spring, MD, U.S.A.

**Keywords:** Chagas disease vector, *Triatomadimidiata* s.l., *
Trypanosoma cruzi
*

## Abstract

A new species of the genus *Triatoma* Laporte, 1832 (Hemiptera, Reduviidae) is described based on specimens collected in the department of Huehuetenango, Guatemala. *Triatomahuehuetenanguensis***sp. n.** is closely related to *T.dimidiata* (Latreille, 1811), with the following main morphological differences: lighter color; smaller overall size, including head length; and width and length of the pronotum. Natural *Trypanosomacruzi* (Chagas, 1909) infection, coupled with its presence in domestic habitats, makes this species a potentially important vector of *Trypanosomacruzi* in Guatemala.

## Introduction

As of 2010, more than a million cardiomyopathy cases in Latin America were caused by Chagas disease (World Health Organization 2015) due to the parasite *Trypanosomacruzi* (Chagas, 1909). This illness is mainly transmitted through the infected feces of insects of the subfamily Triatominae (Hemiptera: Reduviidae). Known colloquially as kissing bugs, the group is currently divided into five tribes and 15 genera (Justi and Galvao 2017; World Health Organization 2015; Schofield and Galvao 2009). Among these, *Triatoma* Laporte, 1832, *Panstrongylus* Berg, 1879 and *Rhodnius* Stål, 1859 (the first two belonging to the tribe Triatomini and the third genera belonging to Rhodniini) are the most epidemiologically relevant for Chagas transmission (WHO 2002).

The genus *Triatoma* is the most diverse, comprising over half of the described Triatominae species (Justi and Galvao 2017; Schofield and Galvao 2009). *Triatomadimidiata* (Latreille, 1811), the most important Chagas disease vector in Central America, is in fact a species complex including at least three independently evolving lineages initially identified by sequences of nuclear (internal transcribed spacer, ITS-2) and the mitochondrial marker cytochrome b (cytb) (Bargues et al. 2008; Dorn et al. 2016), and later confirmed by phylogenetic studies using SNPs and species delimitation, obtained by a reduced representation genome genotyping by sequencing (GBS) approach (Justi et al. 2018). These studies recovered *T.dimidiata* as four linages: groups 1 – 4, which appeared to include at least three species: groups 1 and 2 – *T.dimidiata* s. str., group 3 – Triatomasp. affdimidiata, and group 4 – Triatomasp. affdimidiata cave (Bargues et al. 2008, Dorn et al. 2016, Justi et al. 2018). The last of these taxa, Triatomasp. affdimidiata cave (= group 4) was recently described as a new species, *Triatomamopan* Dorn, Justi & Dale, 2018.

In this study, we formally describe Triatomasp. affdimidiata (group 3) based on morphological and molecular data and name it *Triatomahuehuetenanguensis* sp. n., after the type locality in Guatemala.

## Materials and methods

### Sampling

A total of 39 *Triatoma* specimens was obtained between April 2015 and May 2016 through community participation in the department of Huehuetenango, Guatemala and given to personnel from the Ministry of Health of Huehuetenango who shipped them to the Applied Entomology and Parasitology Laboratory (LENAP), at San Carlos University in Guatemala City. At LENAP the specimens were preserved in 95% ethanol and 5% glycerol and stored at room temperature. Specimens were identified as *T.dimidiata* using the taxonomic key for the genus *Triatoma* published by Lent and Wygodzinsky (1979).

Three females and three males were left intact to comprise the type series used for the morphological description of the new species. DNA was extracted from the remaining 20 by Justi et al. (2018) and tested for infection with *T.cruzi*. Justi et al. (2018) previously recovered all 20 specimens within the same highly supported monophyletic clade named Triatomasp. aff.dimidiata based on genome SNP phylogenies.

### Morphological characterization

Since there is no known holotype for *T.dimidiata* (Latreille, 1811), the characterization of the new species was done following the same methodology as Dorn et al. (2018) for the description of *T.mopan*.

Based on (Lent and Wygodzinsky 1979), 17 morphological traits were measured from the type series and an additional 13 specimens and from 15 female and ten male *Triatomadimidiata* s. str. (group 1–2) from Huehuetenango, Jutiapa and Chiquimula in Guatemala. These 25 *Triatomadimidiata* s. str. specimens were preserved under the same conditions as the new species (95% ethanol and 5% glycerol). Measurements of the morphological traits were performed using a Nikon stereoscope Model SMZ-1B (see Suppl. material 1: morphological measurements). The morphological traits were:

(1) total length,

(2) width of the pronotum,

(3) width of the abdomen,

(4) head length,

(5) width across eyes,

(6) length of the pronotum,

(7) ante-ocular region,

(8) post-ocular region,

(9) width of the eye,

(10) synthlipsis,

(11–14) each of the four antennomeres, and

(15–17) each of the three labial articles.

Because of unequal sample sizes for each group (*T.dimidiata* s. str. and the new species), an unpaired t test was used to compare the means of each of the 17 morphological traits in the two groups (JMP Pro version 13.0.0).

Insects were photographed using a Visionary Digital BK Laboratory System, a Canon 5D camera, 65 mm macro zoom lens. Photo stacks of 25–45 slices were compiled using Helicon Focus 5.3 and the image edited to balance light quality, remove background blemishes, and provide a scale on Photoshop CS6.

### *Trypanosomacruzi* infection

Natural infection by *T.cruzi* was tested by PCR on genomic DNA extracted from the last three segments of the specimens’ abdomen. DNA was extracted using Qiagen DNeasy blood and tissue kit, following the manufacturer’s tissue protocol for the first two steps, blood protocol for subsequent steps and an additional incubation (65 °C for 10 min, followed by 95 °C for 5 min.). Primers and PCR assay conditions were used as previously described (Moser et al. 1989).

### Molecular phylogenetic analysis

In order to: (a) keep the type series intact, (b) confirm that any specimens that share the same phenotype with the type series belong to Triatomasp. affdimidiata, and (c) to determine the relationship with the other groups of *T.dimidiata* s.l., ITS-2 and cytB were sequenced for two out of the 20 Triatomasp. aff.dimidiata specimens studied by Justi et al. (2018). Sequencing was performed as previously described by Dorn et al. (2016). For comparison, ITS-2 and cytB sequences including representatives from all *T.dimidiata* s.l. groups were retrieved from GenBank (Table 1) and aligned using the algorithm Q-INS-I implemented in the online MAFFT version 7 (Katohet al. 2017) and ClustalW (Larkin et al. 2007) implemented on MEGA v. 6 (Tamura et al. 2013), respectively. Maximum likelihood phylogenies were reconstructed independently for each of the genes using PhyML v.3.1 (Guindon and Gascuel 2003), with 100 bootstrap replicates. The best fit model for each gene, according to the AIC criterion, estimated using JModeltest (Darriba et al. 2015), were model HKY+I for ITS-2 and HKY + G for cytB. *Triatomainfestans* (Klug, 1834) was used as the outgroup. Both phylogenies were reconstructed using the same specimens for both markers, as indicated in the original studies. Phylogenies were plotted as mirror images using the function cophyloplot, from the package ape (Paradis et al. 2004), in R (R Development Core Team, 2013). Specimens photos and clade highlights were inserted using Adobe Photoshop CC 2108.

**Table 1.** *Triatoma* specimens used to reconstruct the phylogeny, including collection location, sample identification, and GenBank accession numbers.

Taxon	Locality	Sequence ID	ITS-2	Cyt b
*** T. dimidiata ***	1	Sta. Theresa, Toledo, Belize	DQ871354	FJ197155
	10	Caserío la Bendición, Santa Ana, El Salvador	AM286693	JN585881
	11	Caserío la Bendición, Santa Ana, El Salvador	AM286693	JN585881
	12	Caserío la Bendición, anta Ana, El Salvador	AM286693	JN585881
	13	Caserío la Bendición, Santa Ana, El Salvador	AM286695	JN585881
	14	Caserío la Bendición, Santa Ana, El Salvador	KT874438	JN585881
	15	Sto. Tomás, Sto. Domingo, Heredia, Costa Rica	AM286693	JN585893
	16	Sto. Tomás, Sto. Domingo, Heredia, Costa Rica	AM286693	JN585894
	17	Sto. Tomás, Sto. Domingo, Heredia, Costa Rica	AM286693	JN585894
	18	Angeles, San Rafael, Heredia, Costa Rica	KF192843	JN585894
	19	Sto. Tomás, Sto. Domingo, Heredia, Costa Rica	KT874433	JN585895
	2	Mérida, Yucatán, Mexico	FJ197146	FJ197157
	20	Colombia	AM286703	KT998309
	21	Colombia	AM286703	KT998309
	22	Colombia	AM286704	KT998309
	23	Colombia	KF192845	KT998310
	24	Lanquin, Alta Verapaz, Guatemala	AM286702	KT998313
	25	Lanquin, Alta Verapaz, Guatemala	AM286702	KT998314
	26	El Lodo Negro, Intibuca, Honduras	AM286694	KT998315
	27	El Masical, San Antonio, Copán, Honduras	AM286694	KT998316
	28	El Masical, San Antonio, Copán, Honduras	AM286695	KT998316
	29	Caserío la Bendición, Santa Ana, El Salvador	AM286693	KT998317
	3	Lanquin, Alta Verapaz, Guatemala	AM286694	JN585861
	30	Caserío la Bendición, Santa Ana, El Salvador	AM286696	KT998318
	31	El Lodo Negro, Intibuca, Honduras	AM286695	KT998319
	32	El Masical, San Antonio, Copán, Honduras	AM286694	KT998320
	33	El Lodo Negro, Intibuca, Honduras	AM286693	KT998321
	34	El Lodo Negro, Intibuca, Honduras	KT874435	KT998321
	35	El Lodo Negro, Intibuca, Honduras	KT874437	KT998321
	36	El Masical, San Antonio, Copán, Honduras	AM286693	KT998322
	37	El Masical, San Antonio, Copán, Honduras	KT874436	KT998322
	38	El Lodo Negro, Intibuca, Honduras	AM286693	KT998325
	39	El Lodo Negro, Intibuca, Honduras	AM286694	KT998325
	4	Lanquin, Alta Verapaz, Guatemala	AM286702	JN585861
	40	El Lodo Negro, Intibuca, Honduras	AM286695	KT998325
	41	El Masical, San Antonio, Copán, Honduras	KT874434	KT998325
	42	Caserío la Bendición, Santa Ana, El Salvador	AM286693	KT998327
	43	Angeles, San Rafael, Heredia, Costa Rica	AM286693	KT998328
	44	Sto. Tomás, Sto. Domingo, Heredia, Costa Rica	KT874432	KT998330
	45	Angeles, San Rafael, Heredia, Costa Rica	KF192844	KT998331
	46	San Pedro Columbia, Toledo district, Belize	FJ197153	FJ197154
	5	Caserío la Bendición, Santa Ana, El Salvador	AM286693	JN585881
	6	Caserío la Bendición, Santa Ana, El Salvador	AM286693	JN585881
	7	Caserío la Bendición, Santa Ana, El Salvador	AM286693	JN585881
	8	Caserío la Bendición, Santa Ana, El Salvador	AM286693	JN585881
	9	Caserío la Bendición, Santa Ana, El Salvador	AM286693	JN585881
*** T. huehuetenanguensis ***	A10227	Caserío San Pedro, Huehuetenango, Guatemala	MG947606	MG951755
	A10058	Comunidad El Rosario, Huehuetenango, Guatemala	MG947605	MG951754
** T. sp. aff dimidiata **	1	Calla Creek, Cayo District, Belize	FJ197152	FJ197156
	2	Mérida, Yucatán, Mexico	FJ197150	FJ197158
	3	Mérida, Yucatán, Mexico	FJ197147	FJ197159
	4	Teya, Yucatán, Mexico	KT874439	KT998296
*** T. mopan ***	1	Río Frio Cave, Cayo District, Belize	KF192846	JN585883
	2	Río Frio Cave, Cayo District, Belize	KF192847	JN585884
*** T. infestans ***			AJ576054	JN006799

### Distribution map

**Figure 1.**
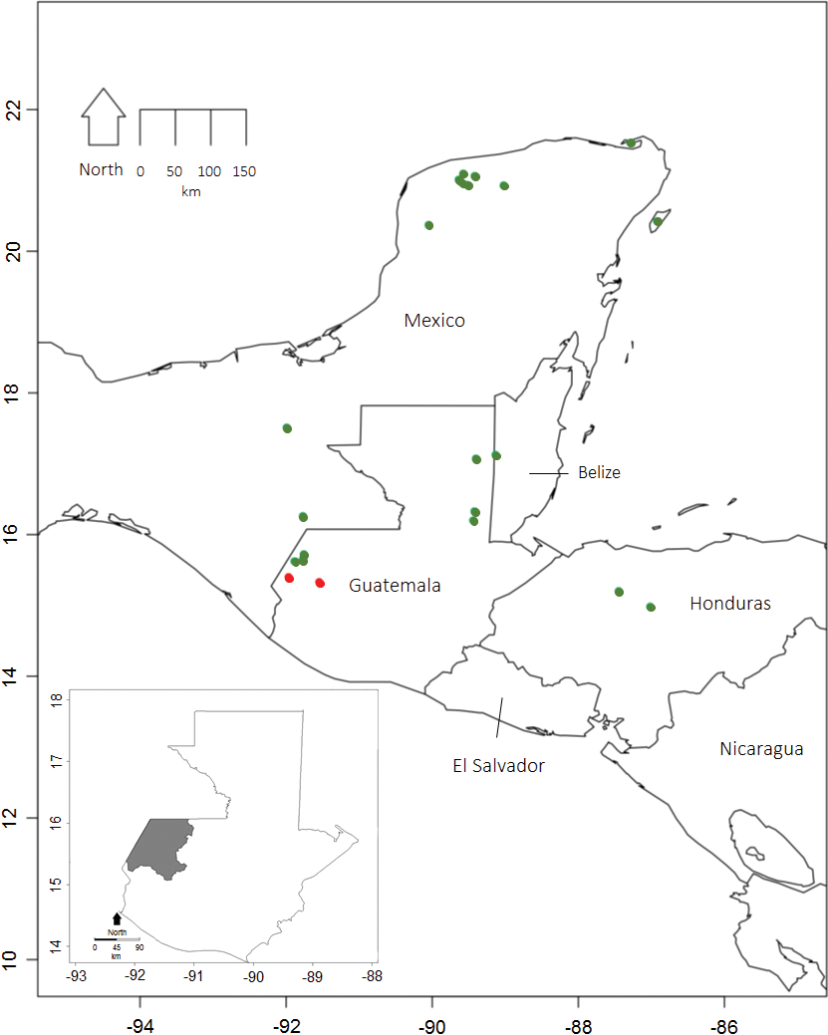
*Triatomahuehuetenanguensis* distribution map based on Bargues et al. (2008), Dorn et al. (2016), Justi et al. (2018) and this study. The red spots designate the places where the holotype and paratypes were collected, the green points refer to the locations where T.sp. aff.dimidiata was previously reported. Map insert highlights the department of Huehuetenango, where *T.huehuetenanguensis* holotype and paratypes were collected.

Reported confirmed distributions of *T.huehuetenanguensis* sp. n. and specimens previously identified as Triatomasp. aff.dimidiata, by molecular means were compiled (Table 2, Fig. 1) and GPS coordinates and altitudes were inferred using Google Maps. Localities were plotted on a map of Central America, using the packages plyr (Wickham 2011), raster (Hijmans 2012) and maps (Becker 2017) available in R (R Development Core Team 2008). The script is available in a supplementary file (Suppl. material 2: Script Huehuetenango map).

**Table 2.** Localities where *T.huehuetenanguensis* (Triatomasp. aff.dimidiata) was reported and corresponding longitude, latitude and altitude (meters above the sea level [m a.s.l.]).

Locality	Longitude	Latitude	Altitude (m a.s.l.)	Reference
**Barrio La Unión, Huehuetenango, Guatemala**	-91.771511, 16.261333		918	Justi et al. 2018
**Calkiní, Campeche, Mexico**	-90.0505156, 20.3707299		16.61	Dorn et al. 2016
**Calla Creek, Cayo District, Belize**	-89.1338271, 17.1257479		69.55	Dorn et al. 2016
**Caserío San Pedro, Cuilco, Huehuetenango, Guatemala**	-91.966667, 15.4		1144.82	This study
**Chablekal, Merida, Yucatan, Mexico**	-89.5756987, 21.0961842		8.9	Bargues et al. 2008
**Chapalá, Cuilco, Huehuetenango, Guatemala**	-91.966667, 15.4		1144.82	This study
**Comunidad El Rosario, Huehuetenango, Guatemala**	-91.47826, 15.31871		1889.08	Justi et al. 2018
	-91.966667, 15.4		1144.82	This study
**Cozumel island, Quintana Roo, Mexico**	-86.9223432, 20.4229839		14.02	Bargues et al. 2008
**El Escondido, La Democracia, Huehuetenango, Guatemala**	-91.887456, 15.622212		895.51	Justi et al. 2018
**El Paraiso, Yoro, Honduras**	-87.016667, 14.983333		1753.10	Bargues et al. 2008
**El Triunfo, Poptún, Petén, Guatemala**	-89.4221967, 16.3279338		521.54	Dorn et al. 2016
	-89.4221967, 16.3279338		521.54	Dorn et al. 2016
**Holbox island, Quintana Roo, Mexico**	-87.2866995, 21.5308421		3	Bargues et al. 2008
**Ixchehuex, Jacaltenango, Huehuetenango, Guatemala**	-91.759167, 15.722778		959.91	Justi et al. 2018
**Izamal, Yucatan, Mexico**	-89.0227126, 20.9299997		14	Bargues et al. 2008
**Las Galeras, San Antonio, Huehuetenango, Guatemala**	-91.771362, 15.651625		1031.98	Justi et al. 2018
	-91.771362, 15.651625		1031.98	Justi et al. 2018
**Los Chucles, La Democracia, Huehuetenango, Guatemala**	-91.88062, 15.62151		895.51	Justi et al. 2018
**Los Encuentros, Poptún, Petén, Guatemala**	-89.4221967, 16.3279338		512.87	Dorn et al. 2016
**Mérida, Yucatán, Mexico**	-89.59258, 20.96737		10.72	Dorn et al. 2016
**Palenque, Chiapas, Mexico**	-91.9930466, 17.5109792		67.08	Bargues et al. 2008
**Paraıso, Yucatan, Mexico**	-89.638845, 21.0108241		9.97	Bargues et al. 2008
**Sabener, Cuilco, Huehuetenango, Guatemala**	-91.966667, 15.4		1144.82	Justi et al. 2018
**San Luis, Petén, Guatemala**	-89.4399388, 16.2000032		381.78	Dorn et al. 2016
**Subirama, Yoro, Honduras**	-87.4480115, 15.2021628		843.60	Bargues et al. 2008
**Teya, Yucatán, Mexico**	-89.5220406, 20.9358411		12.87	Dorn et al. 2016
**Yaxha, Peten, Guatemala**	-89.4024797, 17.0734395		258.80	Bargues et al. 2008
**Yaxkukul, Yucatan, Mexico**	-89.4204, 21.0615692		14.78	Bargues et al. 2008

## Taxonomy

### Family Reduviidae Latreille, 1807

#### Subfamily Triatominae Jeannel, 1919

##### Genus *Triatoma* Laporte, 1832

###### Triatoma huehuetenanguensis

Taxon classificationAnimaliaHemipteraReduviidae

Lima-Cordón & Justi sp. n.

http://zoobank.org/F785B1DC-4946-4FAE-BA5D-D36CA413C67B

####### Material.

**Holotype**: Male. GUATEMALA: Huehuetenango, La Democracia, Aldea Chamuxu, coordinates: 15.6333/-91.8667, 2 May 2016, C. Monroy and A. Rodas, National Museum of Natural History, Smithsonian Institution (voucher: USNMENT01241940). **Paratypes**: One female. GUATEMALA: Huehuetenango, San Pedro Necta, Caserio San Juan, coordinates 15.5/-91.76667, 1 June 2016, C. Monroy and A. Rodas, National Museum of Natural History, Smithsonian Institution, (voucher: USNMENT01241941). Two males. GUATEMALA: Huehuetenango, La Democracia, Aldea Chamuxu, coordinates: 15.6333/-91.8667, 2 May 2016, C. Monroy and A. Rodas, and GUATEMALA: Huehuetenango, San Antonio Huista, Canton Reforma, coordinates: 15.65/-91.7667, May 2016, C. Monroy and A. Rodas, Applied Entomology and Parasitology Laboratory- LENAP (ID: A10723 and A10685, respectively). Two females. GUATEMALA: Huehuetenango, La Democracia, Aldea Chamuxu, coordinates: 15.6333/-91.8667, 28 May 2016, C. Monroy and A. Rodas, and GUATEMALA: Huehuetenango, La Democracia, Aldea Chamuxu, coordinates: 15.6333/-91.8667, 28 May 2016, C. Monroy and A. Rodas, Applied Entomology and Parasitology Laboratory- LENAP (ID: A10800 and A10673, respectively).

####### Etymology.

The name *Triatomahuehuetenanguensis* is in reference to the locality (Department of Huehuetenango, Guatemala) where the holotype and paratype specimens were collected.

####### Differential diagnosis.

Specimens of *T.huehuetenanguensis* are classified as *T.dimidiata* following the key published by Lent and Wygodzinsky (1979). On closer examination, *T.huehuetenanguensis* differs from *T.dimidiata* in the following diagnostic characters: overall color of connexivum, color of head pilosity, ocelli, setae in the second antennomere, anterolateral angles, labial articles joints, setae in the abdomen, spiracles, and female and male terminalia.

**Figure 2.**
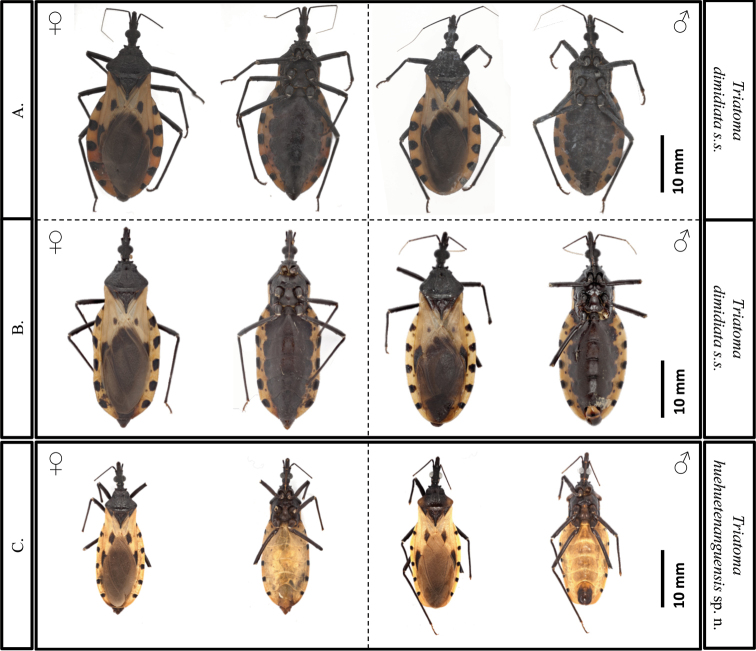
Comparison between *T.dimidiata* s. str. and *T.huehuetenanguensis* sp. n. **A***T.dimidiata* female (left) and male (right) from Jutiapa (dorsal and ventral view) **B***T.dimidiata* female (left) and male (right) from Huehuetenango (dorsal and ventral view) and **C***T.huehuetenanguensis* sp. n. female (left) and male (right) from Huehuetenango (dorsal and ventral view). Photograph credits: RL and SJ.

**Figure 3.**
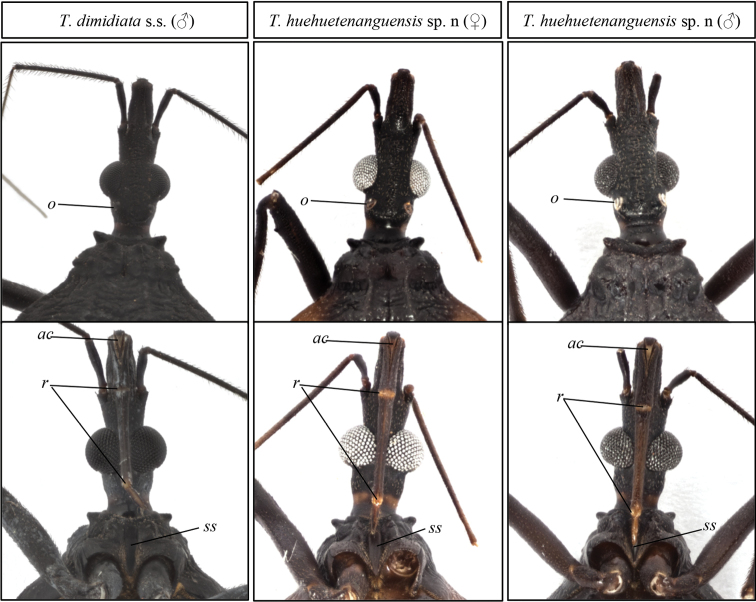
Heads of *T.dimidiata* s. str. and *T.huehuetenanguensis* sp. n. Top panel, dorsal view of the head. Bottom panel, ventral view of the head. Abbreviations: **o** ocelli, **ac** apex of clypeus, **ss** stridulatory sulcus, **r** connections between rostral segments. Photograph credits RL and SJ.

**Figure 4.**
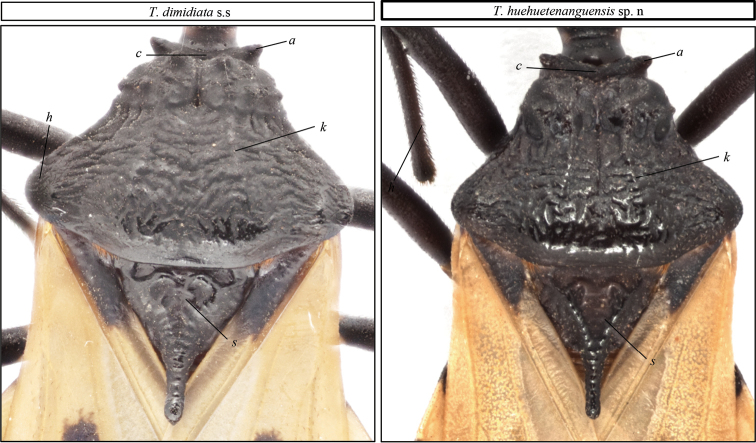
Pronotum of *T.dimidiata* s. str. (left), and *T.huehuetenanguensis* sp. n. (right). Abbreviations: **c** collar, **a** anterolateral angles, **h** humerus and **s** scutellum. Photograph credits RL and SJ.

**Figure 5.**
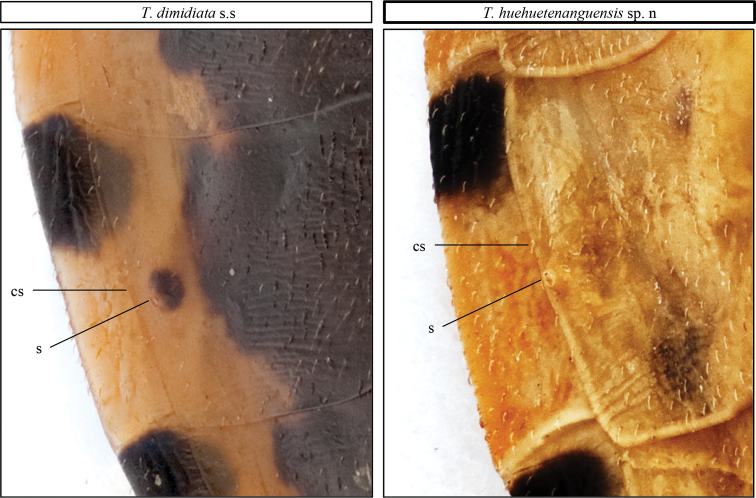
Ventral connexival plate and part of 4^th^ urosternite of *T.dimidiata* s. str. (left), and *T.huehuetenanguensis* sp. n. (right). Abbreviations: **cs** connexival suture and **s** spiracles. Photograph credits RL and SJ.

In contrast to the connexivum and corium color of *T.dimidiata* (pale yellow to orange yellow), *T.huehuetenanguensis* is brown, with connexivum and corium from yellow to pale yellow. The ventral color in *T.huehuetenanguensis* is light yellow, while in *T.dimidiata* it is piceous or black (Fig. 2). The setae around the abdomen are less dense in *T.huehuetenanguensis* when compared to *T.dimidiata*. In *T.huehuetenanguensis*, the spiracles are adjacent to the connexival suture, while in *T.dimidiata*, the spiracles are close but not adjacent to the connexival suture. In addition, spiracles are surrounded by a dark spot in *T.dimidiata*, while the spot is absent in *T.huehuetenanguensis* (Fig. 5). The first antennomere in *T.huehuetenanguensis* does not reach the apex of the head, whereas in *T.dimidiata*, it does. The setae in the second antennomere of *T.huehuetenanguensis* are not as dense as in *T.dimidiata* (Fig. 3). Anterolateral angles are laterally oriented in *T.huehuetenanguensis*, while in *T.dimidiata* they are anterolaterally oriented. The three labial articles of *T.huehuetenanguensis* are light colored while in *T.dimidiata* they are dark, similar to the dark body color. The joint of each of the labial articles are pale yellow only in *T.huehuetenanguensis* (Fig. 3). The collar is relatively thicker in *T.huehuetenanguensis* compared with *T.dimidiata*. The scutellum is rugose in both, *T.dimidiata* and *T.huehuetenanguensis*. However, the central scutellum area in *T.huehuetenanguensis* is more depressed as compared with *T.dimidiata* (Fig. 4). Legs in *T.huehuetenanguensis* with 1 + 1 subapical denticles, sometimes with a very small apical denticle on fore and/or mid-femora. Females sometimes with only one subapical denticle or a callosity, slightly lighter than the tegument on its proximal side. Fore and mid-femora of both males and females with lighter ventral subapical band, ranging from almost imperceptible to yellow.

**Figure 6.**
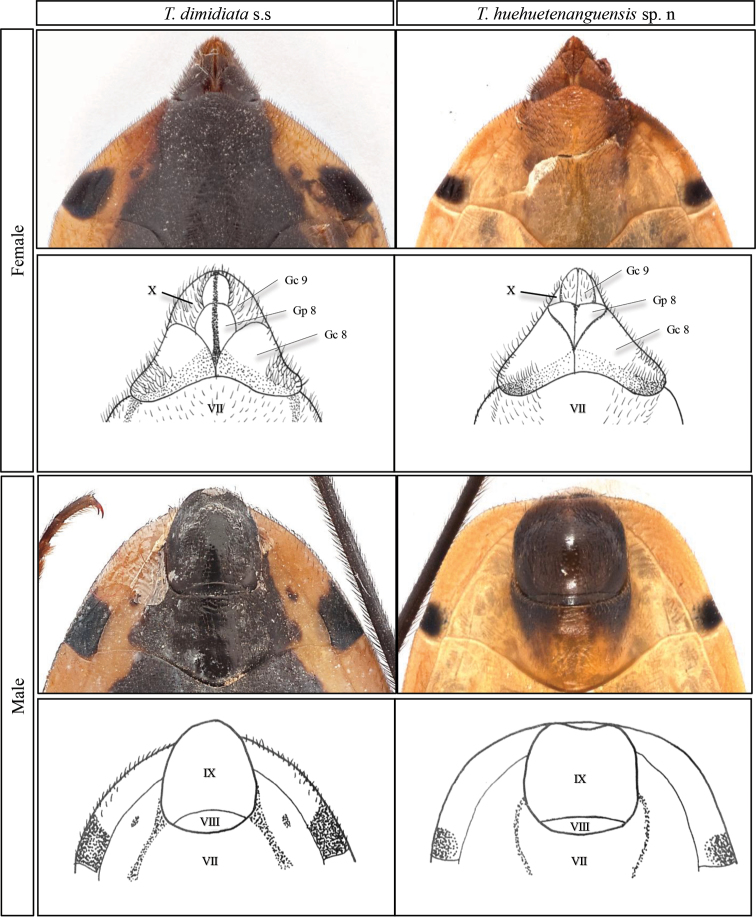
Comparison between the external terminalia of *T.dimidiata* s. str. and *T.huehuetenanguensis* sp. n. Abbreviations: **Gc 8** gonocoxite VIII; **Gc 9** gonocoxite IX; **Gp8** gonapophysis VIII; **VII** sternite; **IX** and **X** segments. Drawings RL. Photograph credits RL and SJ.

The terminalia in males of *T.huehuetenanguensis* is almost square-shaped and darker than the rest of the tegument, presenting sparse dark pilosity, while in *T.dimidiata* it is ovoid and dark, presenting abundant dark pilosity. Posterior margin of urosternite VIII convex on *T.dimidiata* and almost straight in *T.huehuetenanguensis*. Posterior margin of urosternite IX slightly sinuous and not exceeding the abdomen on *T.huehuetenanguensis*, convex and exceeding the abdomen on *T.dimidiata*. Female terminalia in both species is triangle-shaped with very dark and dense pilosity. However, in *T.huehuetenanguensis* it is pale and very pointed while, in *T.dimidiata* it has rounded apex and is dark colored. Posterior margin of sternite VII sinuous on *T.dimidiata* and very sinuous in *T.huehuetenanguensis*; gonocoxite VIII (Gc8) pointed on *T.huehuetenanguensis* and rounded on *T.dimidiata*; gonapophysis VIII (Gp8) is wider than long in *T.huehuetenanguensis* compared to *T.dimidiata*. Gonocoxite IX (Gc9) strongly expanded exceeding the abdomen in *T.huehuetenanguensis* compared to *T.dimidiata* (Fig. 6).

####### Description.

Overall color brown, connexivum, and corium yellow to light yellow. Pilosity short, distinctively yellow, covering whole body, except male and female terminalia, where pilosity is brown.

Total length, male 22.5–26.5 mm, female 22.2–29.3 mm; pronotum width, male 4.9–6.2 mm, females 4.9–6.4 mm; pronotum length, male 3.7–4.2 mm, female 3.4–4.5 mm (Table 3).

**Table 3.** Means and standard deviation (in parenthesis) of 17 morphological traits taken from *T.huehuetenanguensis* sp. n. and *T.dimidiata*.

Morphological character		* T. dimidiata *		* T. huehuetenanguensis *	
		♀ (mm)	♂ (mm)	♀ (mm)	♂ (mm)
**Total length^†^**		33.7 (1.42)	32.6 (0.97)	26.2 (2.29)	25.2 (1.23)
**Width of pronotum^†^**		7.4 (0.51)	7.5 (0.45)	5.7 (0.42)	5.7 (0.39)
**Width of abdomen^†^**		12.8 (1.31)	11.9 (1.03)	8.7 (0.97)	8.4 (0.54)
**Head length^†^**		5.4 (0.33)	5.3 (0.19)	4.5 (0.33)	4.5 (0.18)
**Width across eyes**		2.5 (0.23)	2.6 (0.10)	2.2 (0.15)	2.1 (0.10)
**Length of pronotum^†^**		4.9 (0.32)	5.1 (0.25)	3.9 (0.39)	3.9 (0.16)
**Ante ocular region**		3.0 (0.25)	2.8 (0.08)	2.5 (0.07)	2.3 (0.14)
**Post ocular region**		0.8 (0.13)	0.8 (0.08)	0.8 (0.06)	0.8 (0.10)
**Width of eye**		0.7 (0.11)	0.8 (0.04)	0.6 (0.07)	0.6 (0.07)
**Synthlipsis**		1.0 (0.09)	0.9 (0.06)	0.9 (0.11)	0.9 (0.10)
**Antennae**	1^st^ antennomere	1.4 (0.14)	1.4 (0.11)	0.9 (0.08)	1.1 (0.08)
	2^nd^ antennomere	4.4 (0.42)	4.8 (0.36)	4.0 (0.46)	4.3 (0.16)
	3^rd^ antennomere	3.8 (0.16)	3.6 (0.27)	3.6	3.3
	4^th^ antennomere	3.0 (0.31)	3.0 (0.30)	0.0	2.8
**Labium**	1^st^ article	1.9 (0.23)	1.8 (0.17)	1.9 (0.14)	1.8 (0.24)
	2^nd^ article	3.5 (0.17)	3.5 (0.25)	2.8 (0.20)	2.8 (0.17)
	3^rd^ article	0.97 (0.05)	0.9 (0.11)	0.8 (0.03)	0.9 (0.10)
	**n**	**15**	**10**	**8**	**11**

^†^Statistically significant difference between *T.huehuetenanguensis* and *T.dimidiata* s. str. (p < 0.01, unpaired t-test). Standard deviation is in parenthesis.

Head dark brown with scarce yellow pilosity and overall smooth surface; central band with very shallow rugosity. Head length, male 4.1–4.7 mm, female 4.0–4.8 mm. Ocelli large, lighter than tegument, placed on a pronounced tubercle. Antenniferous tubercles subcylindrical, very short, situated in the middle of the anteocular region; first antennomere not attaining apex of head. Second antennomere lighter than first, with long setae. Ratio of antennomeres I–IV 1:3.9-4.4:3-4:2.5. Apex of clypeus distinctively lighter than rest of head. Labium (Fig. 3) slender, first article reaching level of apex of antenniferous tubercle; second article exceeding posterior border of head, attaining neck; third article light brown, with interarticular areas light yellow (Fig. 3). Ratio of labial articles 1: 1.6:0.5. Most labial setae short, not very numerous on first and second article. Third labial article reaching first third of stridulatory sulcus on males, half on females. Neck dark brown, with very smooth surface, and a pair of lateral yellowish spots.

Pronotum brown, with humerus blunt and pointed, usually lighter in color. Anterolateral angles short, almost round, laterally oriented, almost perpendicular to neck. Submedian carinae very pronounced, with tubercle aspect. Scutellum triangular, shallowly rugose, with the central area distinctly depressed, apical process sometimes lighter in color. (Fig. 4).

Hemelytra not reaching apex of the abdomen, darker at membrane, with scarce light yellow pilosity, dark brown spots around the intersection of the claval suture and the cubital vein, and two larger dark brown spots: one covering the posterior portion of the cubital and medial veins, and the other (largest) covering the joining of the radial and subcostal veins.

Abdomen ventrally convex, shortly pilose, yellow to light yellow (Fig. 3). Spiracles adjacent, but not touching connexival suture, not very pronounced, concolorous with rest of tegument. Connexival segments with a piceous or black spot covering the entire width of the anterior third, yellow posteriorly (Fig. 5).

Males terminalia almost square-shaped, darker than the rest of the body, with scarce dark pilosity. The posterior margin of urosternite VIII almost straight. Posterior margin of urosternite IX slightly sinuous and not exceeding the abdomen. Female external terminalia triangle-shaped, pale, with very dark, dense pilosity (Fig. 6). Posterior margin of sternite VII very sinuous; gonocoxite VIII (Gc8) pointed; gonapophysis VIII (Gp8) is wider than long. Gonocoxite IX (Gc9) strongly expanded exceeding the abdomen in *T.huehuetenanguensis* (Fig. 6).

####### Distribution.

Holotype and paratypes specimens of *T.huehuetenanguensis* were obtained by community participation and reported to be found in domestic environments, near to tropical forest. Huehuetenango is at the northwest of Guatemala and is characterized by pine forest. The altitude ranges from 300 to >3,000 m above sea level. Other localities (Table 1 and Fig. 1) were inferred from specimens collected for previously published molecular phylogenetics studies (Bargues et al. 2008, Dorn et al. 2016, Justi et al. 2018).

####### Host-parasite data.

18 out of the 20 specimens tested were found to be infected with *Trypanosomacruzi*.

####### Discussion.

The ecological diversity within the subfamily Triatominae (>150 species) and its wide distribution through the Americas, and particularly Latin America, have made it difficult to control vector-borne transmission of Chagas disease (WHO 2002). *Triatomadimidiata* is one of the main vector taxa involved in Chagas transmission in Latin America, specifically the main vector for Central America and a secondary vector in Mexico and Colombia. Its broad geographic range and phylogenetic diversity have posed taxonomic challenges for many years (Dorn et al. 2007). Therefore, understanding the taxonomy, phylogenetic and ecological diversity of the *T.dimidiata* complex is important for understanding *T.cruzi* transmission.

Here we are presenting three lines of evidence that support *T.huehuetenanguensis* as a distinct species: morphological, nuclear genetic (ITS-2) and mitochondrial genetic (cytB). The morphological characters included in the taxonomic key for *Triatoma* species (Lent and Wygodzinsky 1979) for *T.dimidiata* encompass *T.huehuetenanguensis*, specifically overall size and measurements of the head and eyes. However, on closer examination, macroscopic differences, including those summarized in Table 4, reveal *T.huehuetenanguensis* as morphologically different. Color differentiation on connexivum, pilosity, ocelli, labial articles joints, and female and male terminalia separate *T.huehuetenanguensis* from *T.dimidiata*. Differentiation based on the color pattern of the connexivum and other body regions was used in the description of *T.brailovskyi* (Martinez, Carcavallo & Pelaez, 1984), *T.gomeznunezi* (Martinez, Carcavallo & Jurberg, 1994) and most recently *Triatomamopan* (Dorn et al., 2018). This latter study attributed the diminished pigmentation of *T.mopan* as the result to the cave environment. In our case, where both, *T.huehuetenanguensis* and *T.dimidiata* are found in sympatry and in domestic environments, it would be interesting to determine the process that caused such color differentiation between these two species. Another important morphological character is the location and pigmentation of the spiracles. These structures in *T.huehuetenanguensis* are adjacent to the connexival suture, whereas in *T.dimidiata*, they are close but not adjacent to the connexival suture. Differentiation based on the location and color of the respiratory spiracles was reported in the description of *Rhodniusmontenegrensis* (Rosa et al., 2012) and most recently in the description of *Triatomamopan* (Dorn et al., 2018).

**Table 4.** Ten distinguishing features between *T.dimidiata* and *T.huehuetenanguensis* sp. n.

Feature	* Triatoma dimidiata * ^†^	*Triatomahuehuetenanguensis* sp. n
**Connexivum overall color**	Dorsal part is pale yellow to orange yellow, and the ventral part is brown to black	Dorsal and ventral sides are yellow to light yellow
**Color of head pilosity**	Dark colored	Light yellow
**Ocelli**	Dark colored	Light colored, implanted in a very pronounced tubercle
**Setae in the 2^nd^** antennomere	Abundant	Scarce
**Anterolateral angles**	Anteriorly oriented, short and sub conical	Laterally oriented, almost perpendicular to the neck, very short and rounder
**Labial articles**	Joint dark colored	Joint yellow colored
**Setae in the abdomen**	Abundant and dark colored	Scarce and yellow colored
**Spiracles**	Close but not touching the connexival suture. Surrounded by a dark spot on the tegument.	Adjacent, but not touching the connexival suture. Same color as the tegument.
**Female external terminalia**	Triangle-shaped with rounded apex	Triangle-shaped with pointed apex
**Male external terminalia**	Ovoid shape	Square-shaped
	Limit of the 8^th^ urosternite is curved	Limit of the 8^th^ urosternite is straight

**^†^***T.dimidiata* features are based on Lent and Wygodzinsky (1979) and *T.dimidiata* s. str. (group 1–2) specimens from Jutiapa, Guatemala.

**Figure 7.**
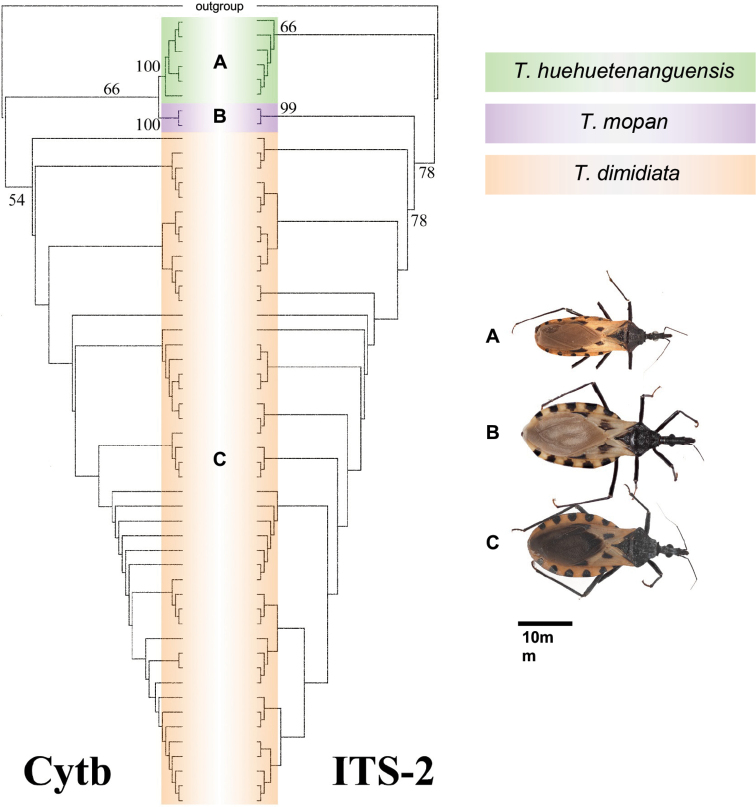
Maximum likelihood cytB and ITS-2 phylogenies. Bootstrap support values of the relevant clades are shown. Habitus of *T.huehuetenanguensis* and related species are shown to scale (10 mm).

The phylogenetic molecular analysis from the nuclear ITS-2 and the mitochondrial cytB gene, recovered a single monophyletic clade with high support (Fig. 7), and low genetic divergence (<3.1% for cytb and < 0.6% for ITS-2) indicating that *Triatomadimidiata* auct., non (Latreille, 1811), Triatomasp. aff.dimidiata (Bargues et al. 2008), Triatomasp. affdimidiata group 3 (Monteiro et al. 2013, Dorn et al. 2016, Justi et al. 2018) and *T.huehuetenanguensis* are the same species (see Suppl. material 3: cytB maximum likelihood phylogeny and Suppl. material 4: ITS-2 maximum likelihood phylogeny). Previously published molecular phylogenies (Bargues et al. 2008, Monteiro et al. 2013, Dorn et al. 2016, Justi et al. 2018) have thoroughly described the ITS-2 and cybB diversity within and among the four groups that comprise *T.dimidiata* s. l. This allowed us to compare these two regions in two of the specimens used by Justi et al. (2018), and verify that they fall within the monophyletic clade. Two Triatominae species were differentiated based on molecular analysis of the mitochondrial gene (Cytb), *R.montenegrensis* (Da Rosa et al. 2012) and *Rhodniusmarabaensis* (Souza et al. 2016).

As supported by our genetic data, we suggest the inclusion of *T.huehuetenanguensis* in the subcomplex *T.dimidiata*. Based on Justi and Galvao (2017), this subcomplex is comprised by: *T.dimidiata*, *T.hegneri*, *T.brailovskyi* and *T.gomeznunezi*, and more recently *T.mopan* has been added (Dorn et al. 2018). The most relevant differences between these six species are summarized in Table 5.

**Table 5.** Distinguishing features between the species of *T.dimidiata* subcomplex based on Justi and Galvão (2017). This reference was used as the original description is not very detailed, and Lent and Wygodzinsky (1979) provide a much more detailed description of the morphology upon inspection of 160 specimens.

Species	Reference	Features
* T. dimidiata *	Lent and Wygodzinsky (1979)*	First antennae segment attaining level of apex of clypeus; anterolateral angles anterolaterally directed; central area of the scutellum not depressed; spiracles close but not adjacent to connexival suture, connexivum dark.
* T. hegneri *	Lent and Wygodzinsky (1979)	Labium very short; abdomen flattened below with spiracles distant from connexival suture; venter and connexivum uniformly dark.
* T. brailovskyi *	Martinez et al. (1984)	Overall size small with very large eyes and ocelli, pronotum with an evident keel at the border, anterolateral angles short and subconical, fore and mid femora with 1 + 1 subapical denticles.
* T. gomeznunezi *	Martinez et al. (1994)	Antenniferous tubercle laterally covered with long setae and dorsally glabrous; neck polished and entirely black; corium dark brown with two basal and distal yellowish spots; venter convex but longitudinally flattened; venter black.
* T. mopan *	Dorn et al. (2018)	Pronotum without discal tubercles and presenting a straight latitudinal depression dividing it in half, fore-femora with 1+1 apical, small denticles, 2 +1 subapical denticles in both males and females; and 1+1 apical, small denticles, 2 +2 asymmetrical subapical larger denticles on males and 2 +2 larger, asymmetrical subapical denticles on females, and spiracles close adjacent to connexival suture, surrounded by a spot slightly darker then the tegument
* T. huehuetenanguensis *	This study	Short yellow pilosity in the whole body except in the genitalia; connections between each segment of the labium are very visible and light-yellow colored; color of venter light yellow.

Relevant Triatominae species for human *T.cruzi* transmission are those that have evolved to live close to humans and have been found to be infected *T.cruzi* (WHO 2002). *Triatomahuehuetenanguensis* was collected in both peridomestic and intradomestic environments. The high natural infection with *T.cruzi* (> 90% of the specimens) suggests it is a potentially important vector and its role in human Chagas disease should be further evaluated.

## Supplementary Material

XML Treatment for Triatoma huehuetenanguensis

## Supplementary materials

Supplementary material 1 Morphological measurements Data type: species data This is an open access article distributed under the terms of the Creative Commons Attribution License (CC BY 4.0), which permits unrestricted use, distribution, and reproduction in any medium, provided the original author and source are credited.Raquel Asunción Lima-Cordón, María Carlota Monroy, Lori Stevens, Antonieta Rodas, Gabriela Anaité Rodas, Patricia L. Dorn, Silvia A. Justi

Supplementary material 2 Script Huehuetenango map Data type: occurrence This is an open access article distributed under the terms of the Creative Commons Attribution License (CC BY 4.0), which permits unrestricted use, distribution, and reproduction in any medium, provided the original author and source are credited.Raquel Asunción Lima-Cordón, María Carlota Monroy, Lori Stevens, Antonieta Rodas, Gabriela Anaité Rodas, Patricia L. Dorn, Silvia A. Justi

Supplementary material 3 CytB maximum likelihood phylogeny Data type: phylogeny data This is an open access article distributed under the terms of the Creative Commons Attribution License (CC BY 4.0), which permits unrestricted use, distribution, and reproduction in any medium, provided the original author and source are credited.Raquel Asunción Lima-Cordón, María Carlota Monroy, Lori Stevens, Antonieta Rodas, Gabriela Anaité Rodas, Patricia L. Dorn, Silvia A. Justi

Supplementary material 4 ITS2 maximum likelihood phylogeny Data type: phylogeny data This is an open access article distributed under the terms of the Creative Commons Attribution License (CC BY 4.0), which permits unrestricted use, distribution, and reproduction in any medium, provided the original author and source are credited.Raquel Asunción Lima-Cordón, María Carlota Monroy, Lori Stevens, Antonieta Rodas, Gabriela Anaité Rodas, Patricia L. Dorn, Silvia A. Justi
