# Supplementary material for: Description of Triatomahuehuetenanguensis sp. n., a potential Chagas disease vector (Hemiptera, Reduviidae, Triatominae)
Source: Zookeys. 2019 Jan 28;(820):51–70. doi: 10.3897/zookeys.820.27258 (PMC6361876; doi:10.3897/zookeys.820.27258)
Supplement: Supplementary material 2 [file zookeys-820-051-s002.pdf]

Triatoma huehuetenanguensis localities

Lori Stevens/Silvia Justi

1/4/2018

Packages used

```
library(lattice)
library(permute)
library(vegan)
```

```
## This is vegan 2.4-4
```

```
library(MASS)
library(sp)
library(foreign)
library(grid)
library(maps)
library(ggplot2)
library(mapdata)
library(dismo)
```

```
## Loading required package: raster

##
## Attaching package: 'raster'

## The following objects are masked from 'package:MASS':
##
##     area, select
```

```
library(maptools)
```

```
## Checking rgeos availability: TRUE
```

```
library(plyr)
```

```
##
## Attaching package: 'plyr'

## The following object is masked from 'package:maps':
##
##     ozone
```

```
library(caper)
```

```
## Loading required package: ape

##
## Attaching package: 'ape'

## The following objects are masked from 'package:raster':
##
##     rotate, zoom

## Loading required package: mvtnorm
```

```
library(RColorBrewer)
library(GISTools)
```

```
## Loading required package: rgeos
## Warning: package 'rgeos' was built under R version 3.4.2
## rgeos version: 0.3-25, (SVN revision 555)
##  GEOS runtime version: 3.6.1-CAPI-1.10.1 r0
##  Linking to sp version: 1.2-5
##  Polygon checking: TRUE
##
## Attaching package: 'GISTools'
## The following object is masked from 'package:maps':
##
##     map.scale
```

Load file with location data. Longitude –>x-axis, latitude → y axis

```
Tria_coords<-read.csv("map_X_phylogeny.csv", header=T)
```

Summarize data by locality

```
b<-ddply(Tria_coords,.(Locality),summarize,Lon=mean(Lon),Lat=mean(Lat))
sp_aff<-b[c(1:4,6,9:28),]
types<-b[c(5,7,8),]
```

Plot the points and call the map with the countries, include the scale and coutry names

```
plot(types$Lon,types$Lat, xlab="",ylab="",xlim=c(-95, -85), ylim=c(10,23),col='red',cex=.8,pch=19)
map("worldHires",c("USA","Mexico","Guatemala", "Belize", "Honduras", "El Salvador", "Costa Rica", "Nica
points(sp_aff$Lon,sp_aff$Lat, xlab="",ylab="",xlim=c(-95, -85), ylim=c(10,23),col='green',cex=.8,pch=19
points(types$Lon,types$Lat, xlab="",ylab="",xlim=c(-95, -85), ylim=c(10,23),col='red',cex=.8,pch=19)

maps::map.scale(-94, 22,ratio=FALSE, relwidth = .1,cex=0.5)
text(-90, 19, "Mexico", cex=0.7)
text(-90.5, 15.3, "Guatemala", cex=0.7)
text(-87.8, 16.9, "Belize", cex=0.7)
text(-89, 12.7, "El Salvador", cex=0.7)
text(-86.2, 15, "Honduras", cex=0.7)
text(-85.5, 13, "Nicaragua", cex=0.7)
north.arrow(xb=-94.5, yb=21.5, len=0.2, lab="N", cex=.7)
```

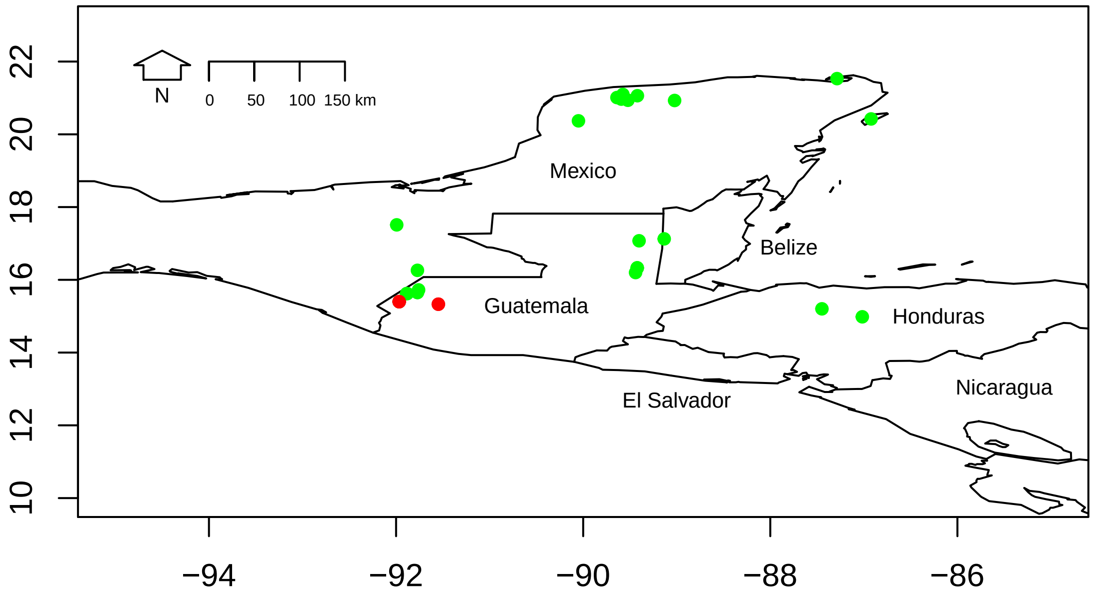
N
0
50
100
150 km
Mexico
Belize
Guatemala
Honduras
Nicaragua
El Salvador
22
20
18
16
14
12
10
−94
−92
−90
−88
−86
