# Supplementary figures and images for: Description of Triatomahuehuetenanguensis sp. n., a potential Chagas disease vector (Hemiptera, Reduviidae, Triatominae)
Source: Zookeys. 2019 Jan 28;(820):51–70. doi: 10.3897/zookeys.820.27258 (PMC6361876; doi:10.3897/zookeys.820.27258)

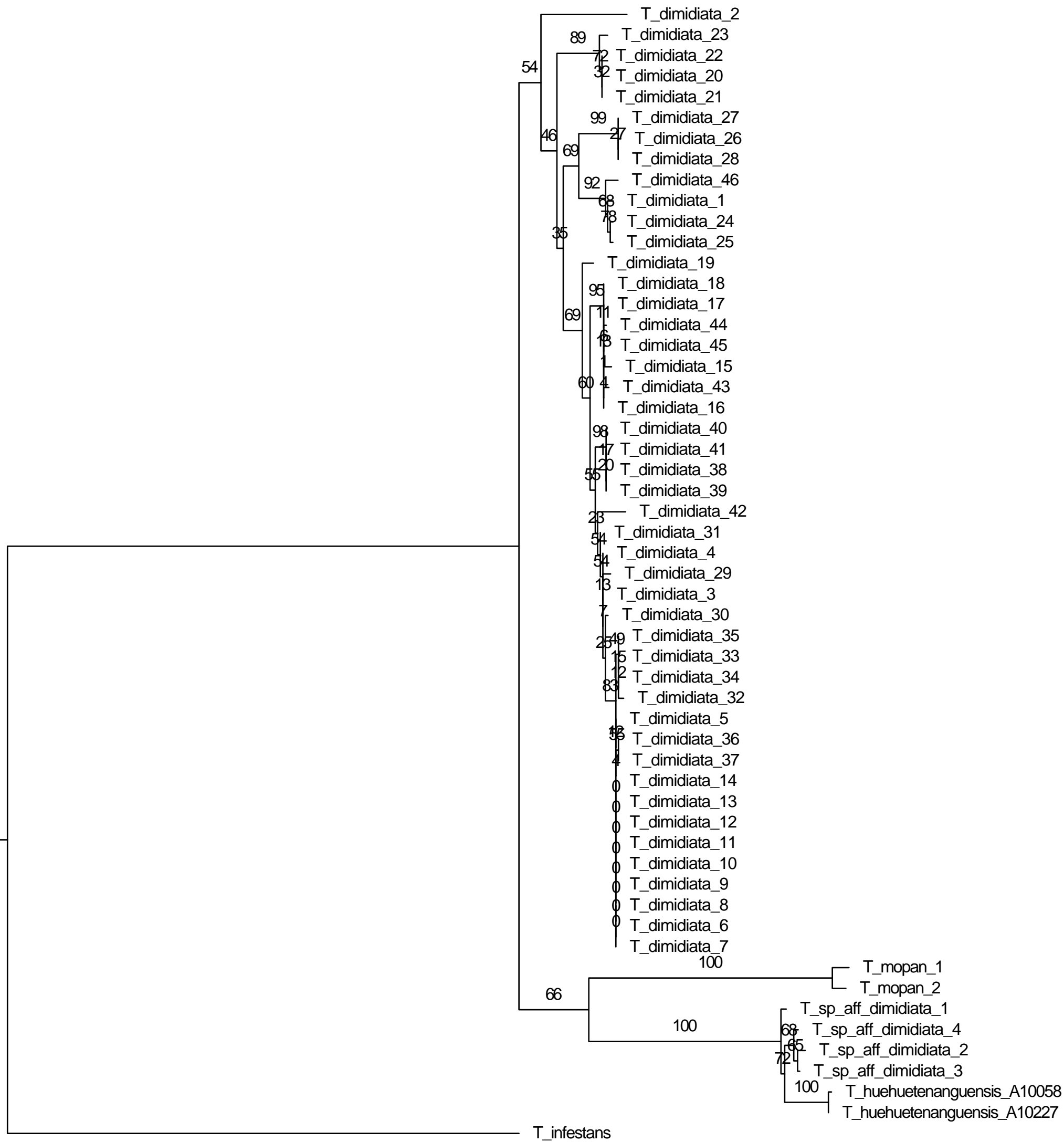

0.09

Supplement: Supplementary material 3 [file zookeys-820-051-s003.pdf]

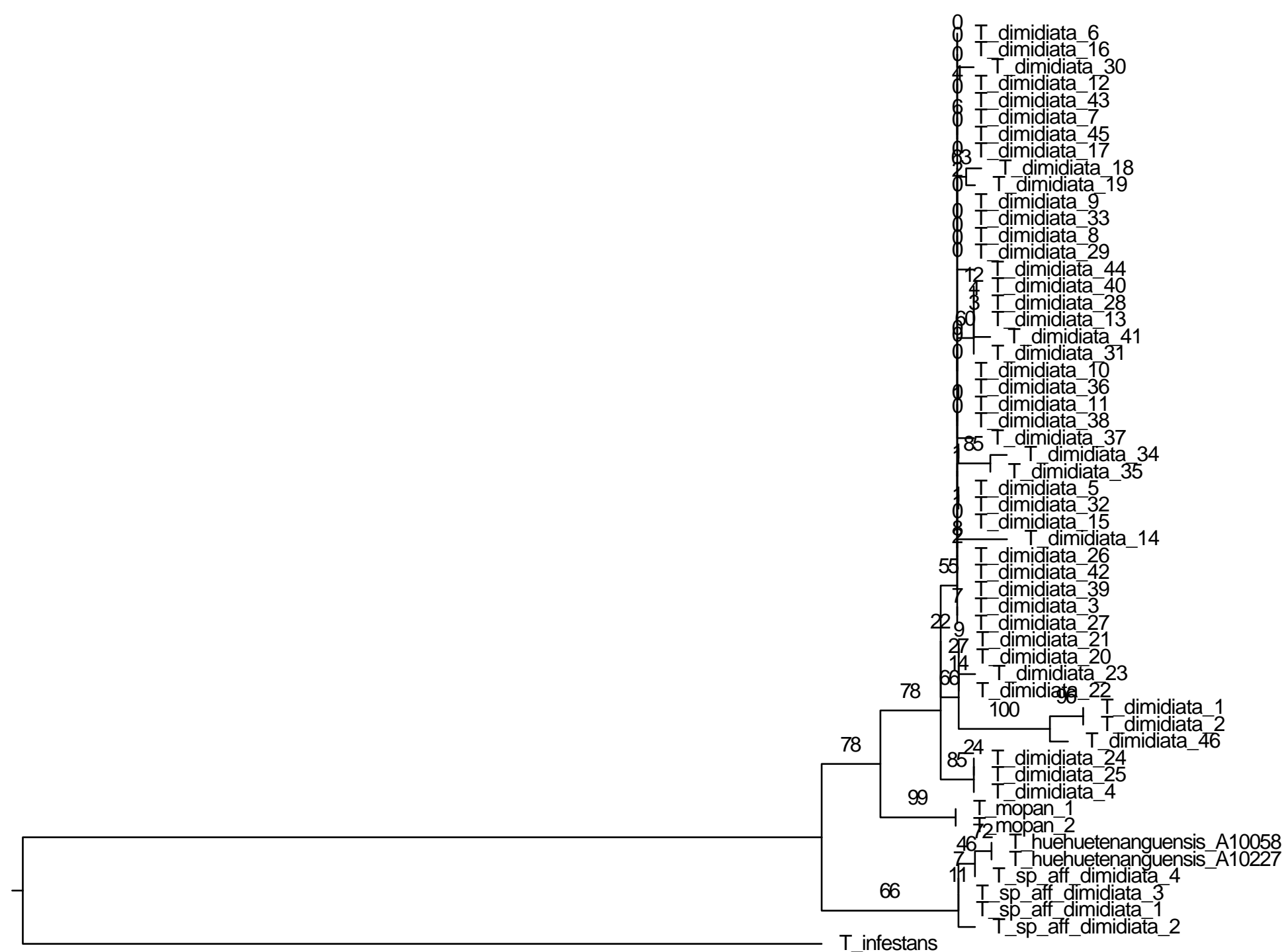

Supplement: Supplementary material 4 [file zookeys-820-051-s004.pdf]
